# Novel PNKP mutations causing defective DNA strand break repair and PARP1 hyperactivity in MCSZ

**DOI:** 10.1212/NXG.0000000000000320

**Published:** 2019-03-25

**Authors:** Ilona Kalasova, Hana Hanzlikova, Neerja Gupta, Yun Li, Janine Altmüller, John J. Reynolds, Grant S. Stewart, Bernd Wollnik, Gökhan Yigit, Keith W. Caldecott

**Affiliations:** From the Department of Genome Dynamics (I.K., H.H., K.W.C.), Institute of Molecular Genetics of the Czech Academy of Sciences, Czech Republic; Genome Damage and Stability Centre (H.H., K.W.C.), School of Life Sciences, University of Sussex, Falmer, Brighton, UK; Institute of Human Genetics (Y.L., B.W., G.Y.), University Medical Center Göttingen, Germany; Cologne Center for Genomics (J.A.), University of Cologne, Germany; Institute of Cancer and Genomic Sciences (J.J.R., G.S.S.), College of Medical and Dental Sciences, University of Birmingham, UK; and Division of Genetics (N.G.), Department of Pediatrics, All India Institute of Medical Sciences, New Delhi, India.

## Abstract

**Objective:**

To address the relationship between novel mutations in polynucleotide 5'-kinase 3'-phosphatase (PNKP), DNA strand break repair, and neurologic disease.

**Methods:**

We have employed whole-exome sequencing, Sanger sequencing, and molecular/cellular biology.

**Results:**

We describe here a patient with *microcephaly with early onset seizures* (MCSZ) from the Indian sub-continent harboring 2 novel mutations in *PNKP*, including a pathogenic mutation in the fork-head associated domain. In addition, we confirm that MCSZ is associated with hyperactivation of the single-strand break sensor protein protein poly (ADP-ribose) polymerase 1 (PARP1) following the induction of abortive topoisomerase I activity, a source of DNA strand breakage associated previously with neurologic disease.

**Conclusions:**

These data expand the spectrum of *PNKP* mutations associated with MCSZ and show that PARP1 hyperactivation at unrepaired topoisomerase-induced DNA breaks is a molecular feature of this disease.

Mutations in DNA single-strand break repair (SSBR) proteins are associated with hereditary neurologic disease.^1,2^ Recently, using a mouse model in which the SSBR protein Xrcc1 is mutated, we demonstrated that hyperactivation of the SSB sensor protein poly(ADP-ribose) polymerase 1 (PARP1) is a likely source of the neuropathology induced by SSBs.^3^ During SSBR, the enzyme polynucleotide 5′-kinase 3′-phosphatase (PNKP) can employ either DNA 3′-phosphatase and/or DNA 5′-kinase activities to restore ligatable termini at DNA strand breaks, and is recruited to SSBs by interacting with XRCC1 via an amino-terminal fork-head associated (FHA) domain.^4–6^ Strikingly, PNKP mutations are associated with 2 apparently distinct neurologic diseases: *microcephaly with early onset seizures* (MCSZ) and *ataxia with oculomotor apraxia 4*.^7,8^ The former is a neurodevelopmental disease associated with developmental delay, microcephaly, and seizures whereas the latter is a degenerative disease associated with cerebellar atrophy, ataxia, and oculomotor apraxia. Intriguingly, some individuals with PNKP mutations exhibit phenotypic aspects of both disorders, and in some cases can overlap with *Charcot-Marie-Tooth disease*.^9,10^ To explain the molecular basis and phenotypic diversity of PNKP-associated disease will require an understanding of the spectrum of PNKP mutations associated with disease, and of the influence of these mutations on DNA repair. Here, we identify 2 novel disease mutations in PNKP in a patient with MCSZ from the Indian subcontinent. We identify a defect in DNA strand break repair in patient-derived primary fibroblasts and that these MCSZ cells are associated with hyperactivation of the SSB sensor protein, PARP1.

## Methods

### Patient case report

**Figure 1:**
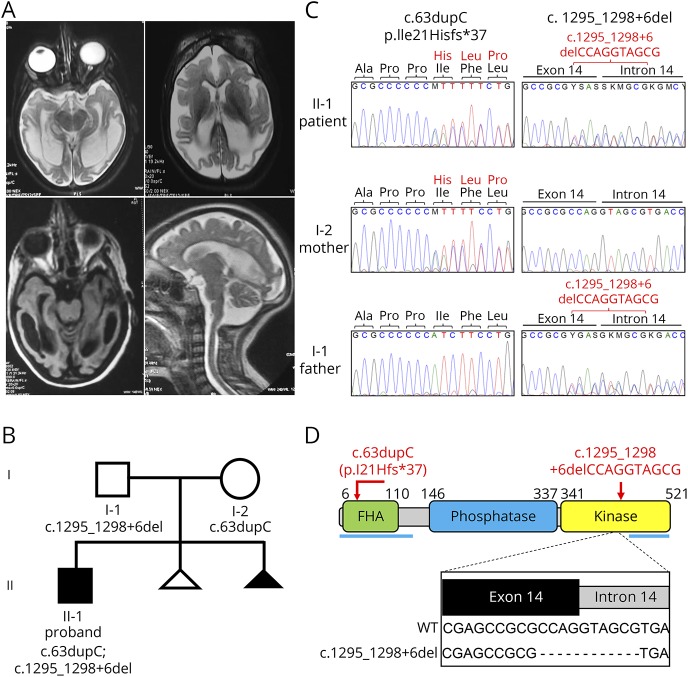
Novel PNKP mutations in an individual from India with MCSZ (A), patient MRI. T2 axial and FLAIR axial images showing microcephaly with diffuse white matter signal changes. There is mild ventriculomegaly with generalized atrophy and cystic changes in bilateral temporal lobes. There is mild hypoplasia of inferior vermis as seen on sagittal T2 weighted image. (B), Pedigree of the proband (black square). The triangles indicate pregnancies that did not go to term. Note that one of these (black triangle) was associated with antenatal detection of microcephaly. (C), Electropherograms showing the compound heterozygous *PNKP* mutations in the proband and heterozygosity in the parents. (D), cartoon of PNKP and its functional domains. The proband mutations, confirmed by Sanger sequencing, are shown in *red*. Epitopes of the N- and C-terminal polyclonal antibodies used in this study are indicated with horizontal *blue lines*. The inset shows the impact of the deletion caused by c.1295_1298+6del on the splice site between exon 14 and intron 14. FLAIR = fluid-attenuated inversion recovery; MCSZ = *microcephaly with early onset seizures*; PNKP = polynucleotide 5′-kinase 3′-phosphatase*.*

The patient is currently a 8.5-year-old boy who was born at term with microcephaly (occipito-frontal circumference 29 cm, –3 SD) and below-normal weight (−2.8 SD). Seizures began at day 15 of his life and he had severe developmental delay. He established head control at 6 months and started sitting with support at month 7, but never attained walking. He developed monosyllable speech at month 8 but since then has exhibited a complete developmental arrest. Examination at ∼year 8.5 revealed severe microcephaly (−14.9 SD), short stature (−6.86 SD), and failure to thrive (weight = −5.83SD). He is a thin child with a slender build, convergent squint, receding forehead, high nasal bridge, bilateral up slant, receding chin, dental crowding and teeth caries, spasticity, brisk reflexes, and striatal toe. Ataxia could not be ascertained. He has a café-au-lait spot on his right upper thigh and dark hyperpigmented skin. He also has a large amplitude stereotype lower limb movements and midline hand stereotypes. Hearing evaluation revealed an absence of waves at 50 dB in the left ear. At year 4.5, T2 axial and fluid-attenuated inversion recovery axial MRI images revealed microcephaly with diffuse white matter signal changes (figure 1A). There was mild ventriculomegaly with generalized atrophy and cystic changes in bilateral temporal lobes. Mild hypoplasia of the inferior vermis was evident on the sagittal T2 weighted image. His CT scan brain did not show any calcifications. EEG at 5 years revealed resolving hypsarryhthmia and evolving multifocal epilepsy. His karyotype, liver function, and renal function were all normal. His serum uric acid was 1.2 mg/dL. His serum α fetoprotein levels are 22.9 ng/mL (normal range 0.89–8.78 ng/mL). His lipid profile showed mildly high triglycerides 161 (normal range 50–150) with low density lipoprotein/high density lipoprotein ratio 3.44. He is at present non-ambulatory, can sit with support, and does not have seizures.

### Whole exome sequencing

Whole-exome sequencing (WES) was performed on DNA extracted from blood lymphocytes of the index patient using the SureSelectXT Human All Exon V6 enrichment kit (Agilent technologies) on an Illumina HiSeq4000 sequencer. In total, we obtained a mean coverage of 84 reads, and 96.3% of target were covered more than 10×. WES data analysis and variant filtering was conducted using the exome analysis pipeline “varbank” of the Cologne Center for Genomics (CCG, varbank.ccg.uni-koeln.de/) and data were filtered for high-quality (coverage of more than 6 reads, a minimum quality score of 10), rare (minor allele frequency < 0.5% in the databases of the exome aggregation consortium and single nucleotide polymorphisms) autosomal recessive variants.

### Mutation screening

Variants identified by WES were amplified from DNA of the index patients, and its parents and PCR products were sequenced by BigDye Terminator method on an ABI 3500 × L Genetic Analyzer (Life Technologies, Germany). Identified mutations were re-sequenced in independent experiments.

### Primary human fibroblasts

Control human fibroblast 1BR3 (denoted 1BR in the text) and PNKP patient-derived skin fibroblasts, obtained with appropriate patient consent, were grown in minimum essential media (Gibco) supplemented with 15% fetal bovine serum, 2 mM glutamine, the antibiotics penicillin (100 units/mL), and streptomycin (100 μg/mL) in a humidified atmosphere of 5% CO_2_ at low oxygen (5%) at 37°C.

### Antibodies, western blotting, and indirect immunofluorescence microscopy

Primary antibodies were anti-pan-ADP-ribose binding reagent (MABE1016, Millipore), anti-PNKP FHA (SK3195), anti-PNKP C-terminal (ab18107, Abcam), anti-p53 (OP29, Millipore), and anti-GAPDH (sc-47724, Santa Cruz). Secondary antibodies were horseradish peroxidase (HRP) conjugated goat anti-rabbit (170-6515, Bio-Rad) and goat anti-mouse (170-6516, Bio-Rad) and for indirect immunofluorescence donkey anti-rabbit Alexa 488 (A21206, Invitrogen). For western blotting, cells were lysed in sodium dodecyl sulphate (SDS) sample buffer and subjected to SDS-PAGE, transferred onto nitrocellulose membrane and detected by specific antibodies combined with HRP conjugated secondary antibodies. For indirect immunofluorescence microscopy, cells were cultured on glass coverslips and treated where indicated with 10 μM camptothecin (CPT) (Sigma) for 45 minutes. Cells were fixed with 4% paraformaldehyde and immunostained as described previously.^3^ Images were taken using a DMi6000 microscope (Leica) with 40x dry objective. Automated wide-field microscopy was performed on ScanR system (Olympus) with ScanR Image Acquisition and Analysis Software, 40x/0.95NA (UPLSAPO 2 40X) dry objective.

### PNKP biochemical activity assays

PNKP substrate was prepared by annealing equimolar amounts of the fluorophore-labeled deoxyriboligonucleotides (Midland Certified Reagent Company) “S1” [5’-(TAMRA)-TAGCATCGATCAGTCCTC-3′-P]**,** “S2” [5′-OH-GAGGTCTAGCATCGTTAGTCA-(6-FAM)-3’] and a complementary strand oligonucleotide [5′-TGACTAACGATGCTAGACCTCTGAGGACTGATCGATGCTA-3’] in annealing buffer (10 mM Tris pH 7.5, 200 mM NaCl, 1 mM EDTA). Cell-free protein extracts were prepared in lysis buffer [25 mM Tris, pH 7.5, 10 mM EDTA, 10 mM EGTA, 100 mM NaCl, 1% Triton X-100, cOmplete protease inhibitors (Roche)] and incubated (25 μg total protein) with 50 nM substrate and 1 μM single-stranded nuclease competitor oligonucleotide [5′-AAAGATCACAAGCATAAAGAGACAGG-3’] in reaction buffer (25 mM Tris, pH 7.5, 130 mM KCl, 10 mM MgCl2, 1 mM DTT, 1 mM ATP) at 37°C for 10 minutes in 50 μL reactions and were terminated by addition of 50 μL quenching buffer (90% formamide, 50 mM EDTA, 0.006% Orange G). 10 μL was separated on 20% denaturing polyacrylamide gels and analyzed on a PharosFX Molecular Imager System (Bio-Rad). Where indicated, cells were transfected with mix of either PNKP siRNA #1: 5′-CCGGAUAUGUCCACGUGAA-3′ and PNKP siRNA #2: 5′-GGAAACGGGUCGCCAUCGA-3′ or non-target siRNA #1: 5′-UGGUUUACAUGUCGACUAA-3′ and non-target siRNA #2: 5′-UGGUUUACAUGUUGUGUGA-3′) using Lipofectamine RNAiMAX (Life Technologies) 72 hours before lysis.

### Cellular DNA strand break assays

The steady-state level of DNA strand breaks was evaluated by alkaline comet assays essentially as described^11^ following mock-treatment or treatment with 10 μM CPT for 60 minutes at 37°C. The average comet tail moment in 100 cells per sample was evaluated by Comet Assay IV software (Perceptive Instruments).

### Standard protocol approvals, registrations, and patient consents

Ethical approval and informed parental consent for the use and publication of patient information and patient derived cell lines was obtained from the appropriate Institutional and regional committees. No vertebrate animal models were employed in this work.

### Data availability

Data will be provided on request at https://sussex.figshare.com/10.25377/sussex.7836653.

## Results

Individuals with neurologic disease resulting from mutations in PNKP protein have been identified in the Americas, Europe, Middle East, and Japan, but surprisingly not yet on the Indian subcontinent. Here, we describe the first Indian patient with biallelic mutations in *PNKP*. The proband is an 8.5 year old boy with *microcephaly, early-onset seizures, and developmental delay* (MCSZ). Seizures were detected from day 15 of life and MRI revealed microcephaly with diffuse white matter signal changes and generalized atrophy in the bilateral temporal lobes (figure 1A). A detailed case report is presented in the Methods.

WES on DNA extracted from blood lymphocytes from the proband identified 2 heterozygous variants in *PNKP* (NM_007254.3); c.63dupC and c.1295_1298+6delCCAGGTAGCG (figure 1B and C). Sanger sequencing confirmed the presence of both mutations in the proband and their heterozygosity in the parents. c.63dupC is a 1 base pair duplication in exon 2 and was inherited from the mother, and c.1295_1298+6delCCAGGTAGCG is a 10 base pair deletion that was inherited from the father (figure 1B and C). The 1 base pair duplication is predicted to cause a frameshift resulting in nonsense-mediated mRNA decay and/or truncated PNKP protein (p.Ile21Hisfs*37) and is the first unambiguously deleterious mutation identified in the FHA domain (figure 1D). The 10 base pair deletion spans the last 4 nucleotides of exon 14 and the first 6 nucleotides of intron 14 (figure 1C) and deletes a donor splice-site, leading most likely to nonsense-mediated mRNA decay and/or a synthesis of a truncated protein.

**Figure 2:**
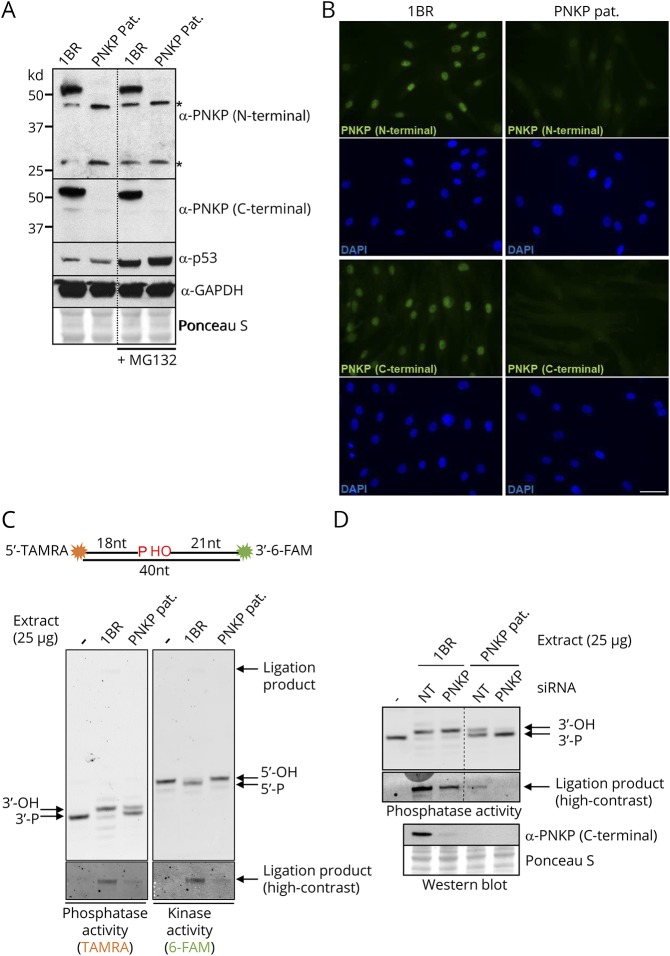
PNKP protein levels and PNKP activity in MCSZ patient-derived fibroblasts (A), western blot showing PNKP protein levels in 1BR and PNKP patient fibroblasts after 6 hours incubation with DMSO vehicle or 10 μM proteasome inhibitor MG132. Two different antibodies were employed, directed at the N- or C-terminus as indicated. p53 and GAPDH detection was employed as a positive control for proteasome inhibition by MG132 and as a loading control, respectively. Asterisks denote nonspecific bands. (B), PNKP indirect immunofluorescence with the antibodies described above in 1BR and PNKP patient fibroblasts. Scale bar 50 μm (C), PNKP DNA 3′-phosphatase and DNA 5′-kinase activity in wild type 1BR and PNKP patient cell extracts. *Top*, A cartoon of the PNKP oligonucleotide duplex substrate, harboring a SSB with 3′-phosphate and 5′-hydroxyl termini. *Bottom*; reaction products of PNKP activity. The positions of the substrate (3′-P) and product (3′-OH) of PNKP 3′-phosphatase activity (*left panel*), and of the substrate (5′-OH) and product (5′-P) of the 5′-kinase activity (*right panel)* are shown. The 40-mer ligation product of the repair reaction, which is labeled with both TAMRA and FAM, is indicated by an arrow in the top panel and is shown in the bottom panels with high contrast (D), PNKP DNA 3′-phosphatase activity in cell extracts from 1BR and PNKP patient fibroblasts additionally transfected with PNKP siRNA. The 40-mer ligation products are highlighted in the middle (high contrast) panel. The PNKP protein levels in the siRNA transfected 1BR and patient fibroblasts are shown at the bottom. MCSZ = *microcephaly with early onset seizures*; PNKP = polynucleotide 5′-kinase 3′-phosphatase; TAMRA = tetramethylrhodamine*.*

To define the consequence of the 2 novel *PNKP* mutations at the molecular and cellular level, we employed primary fibroblasts established from a skin biopsy from the proband. Western blotting using antibodies targeting either the N-terminal or C-terminal regions of PNKP failed to detect PNKP protein in the patient-derived fibroblasts, even if we incubated the cells with the proteasome inhibitor MG132 to prevent the degradation of the mutant PNKP (figure 2A). Incubation with MG132 increased the level of p53 protein, however, confirming that the proteasome was inhibited in these experiments. The impact of the 2 novel mutations on PNKP protein levels was also observed by indirect immunofluorescence, with both N-terminal and C-terminal PNKP antibodies again detecting little or no PNKP in the patient-derived fibroblasts (figure 2B).

Next, we measured the level of PNKP activity using a sensitive biochemical assay.^12^ This assay uses an oligonucleotide duplex substrate possessing an internal single-nucleotide gap with 3′-phosphate and 5′-hydroxyl termini, which is labeled at the ends of the duplex with the fluorescent dyes tetramethylrhodamine and 6-carboxyfluorescein (figure 2C). The 3′-phosphate and 5′-hydroxyl termini in this substrate were completely repaired by incubation with cell extract from wild type fibroblasts within 10 minutes. Although greatly reduced, DNA 3′-phosphatase and 5′-kinase activities were also detected in the patient cell extract as indicated by the formation of a small amount of fully repaired reaction product (“ligation product”) and also in the case of 3′-phosphatase activity by the appearance of a small amount of dephosphorylated oligonucleotide (figure 2C *left panel*,“3′-OH”). This residual activity in the patient-derived fibroblasts was greatly reduced or ablated if the patient cells were first transfected with *PNKP* siRNA, indicating that it reflected residual levels of PNKP (figure 2D). It is currently unclear how the residual PNKP activity is generated in the patient cells, especially with respect to 5′-kinase activity, because both mutations are predicted to result in translational frameshifts and to truncate either almost all of the protein (in the case of the maternal allele, c.63dupC) or a large region of the kinase domain (in the case of the paternal allele, c.1295_1298+6del) (figure 1D). Possible explanations include a low level of alternative splicing, an alternative translation start site downstream of the premature stop codon in the maternal *PNKP* allele, a small amount of properly spliced mRNA arising from the paternal allele, or the presence of a small amount of an alternative 5′-kinase.

**Figure 3:**
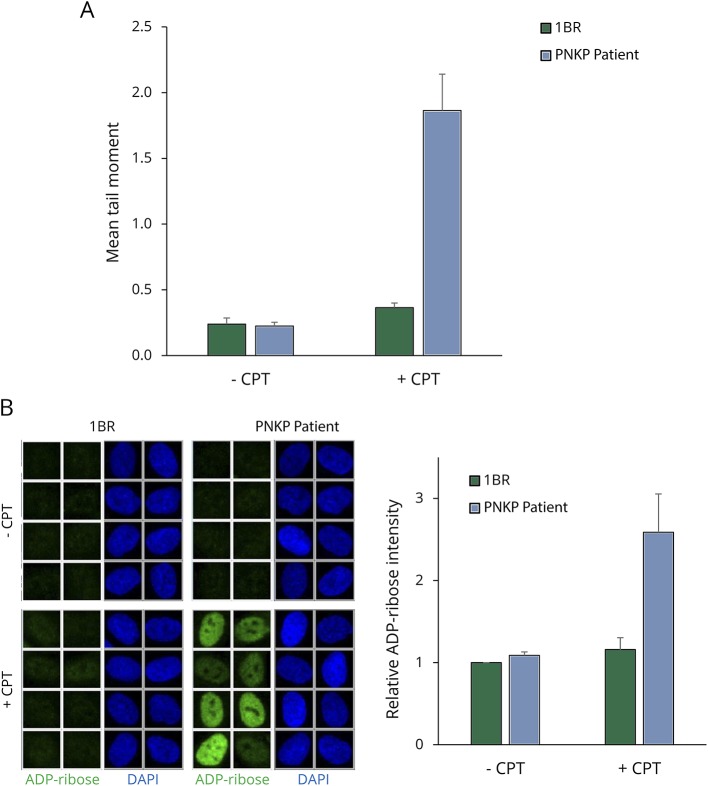
Elevated levels of TOP1-induced DNA breaks and poly(ADP-ribose) in MCSZ fibroblasts (A) The accumulation of CPT-induced DNA strand breaks in 1BR and patient fibroblasts, quantified by alkaline comet assays. Cells were treated with DMSO vehicle or 10 μM CPT for 45 minutes. Data are the average comet tail moment of 100 cells per sample and are the mean (±SEM) of 4 independent experiments. (B) Representative ScanR images (*left*) and quantitation (*right*) of poly (ADP-ribose) levels in control 1BR and PNKP patient fibroblasts incubated in the presence or absence of CPT as above. Data are the mean (±SEM) of 3 independent experiments. CPT = camptothecin; DMSO = dimethyl sulphoxide; MCSZ = *microcephaly with early onset seizures*; PNKP = polynucleotide 5′’-kinase 3′’-phosphatase; SEM = standard error of the mean*.*

We next examined whether the greatly reduced level of PNKP protein and activity in the patient cells affected the rate of DNA strand break repair. DNA strand breaks induced by abortive topoisomerase 1 (TOP1) activity possess termini that are substrates for both the DNA kinase and DNA phosphatase activities of PNKP. Indeed, the PNKP patient fibroblasts accumulated higher levels of DNA strand breaks than 1BR control cells during incubation with TOP1 ‘poison’ CPT (figure 3A). This observation is significant, because defects in the repair of TOP1-induced DNA breaks are also observed in cells from other MCSZ patients and from patients with *spinocerebellar ataxia with axonal neuropathy-1* and *ataxia telangiectasia*, illustrating their relevance to neurodegenerative disease^13–15^*.* Importantly, hyperactivation of the SSB sensor protein PARP1 is observed in and is causally linked with SSBR-defective cerebellar ataxia.^3^ Consequently, we wondered whether PARP1 hyperactivation might also be a feature of these MCSZ cells. Indeed, strikingly, PNKP patient fibroblasts accumulated much higher levels of the poly (ADP-ribose) product of PARP1 activity than did 1BR control fibroblasts during treatment with CPT (figure 3B).

Our data identify 2 novel *PNKP* mutations in a patient with MCSZ from India. Of interest this patient exhibits one of the most severe forms of MCSZ documented, with a level of microcephaly and growth retardation comparable to patients with ataxia telangiectasia and Rad3 related-Seckel Syndrome.^16^ Despite this, in keeping with the functional analysis of other identified PNKP mutations,^15^ cells derived from this patient retain some residual PNKP activity. This is consistent with the observation that complete deletion of PNKP in mouse is embryonic lethal.^17^ Finally, we have confirmed that PARP1 hyperactivation is a cellular hallmark of MCSZ cells, highlighting this phenomenon in the development of a range of clinically distinct neuro-pathologies.

## Glossary

CCG: Cologne Center for Genomics
CPT: camptothecin
FHA: fork-head associated
HRP: horseradish peroxidase
MCSZ: *microcephaly with early onset seizures*
PARP1: protein poly (ADP-ribose) polymerase 1
PNKP: polynucleotide 5′-kinase 3′-phosphatase
SCAN-1: *spinocerebellar ataxia with axonal neuropathy-1*
SSBR: single-strand break repair
WES: whole-exome sequencing
